# Translational research on reserve against neurodegenerative disease: consensus report of the International Conference on Cognitive Reserve in the Dementias and the Alzheimer’s Association Reserve, Resilience and Protective Factors Professional Interest Area working groups

**DOI:** 10.1186/s12916-019-1283-z

**Published:** 2019-02-27

**Authors:** Robert Perneczky, Gerd Kempermann, Amos D. Korczyn, Fiona E. Matthews, M. Arfan Ikram, Nikolaos Scarmeas, Gael Chetelat, Yaakov Stern, Michael Ewers

**Affiliations:** 1 Division of Mental Health in Older Adults and Alzheimer Therapy and Research Center, Department of Psychiatry and Psychotherapy, University Hospital, Ludwig Maximilian University Munich, 80336 Munich, Germany; 2 German Center for Neurodegenerative Diseases (DZNE) Munich, Munich, Germany; 3 Ageing Epidemiology (AGE) Research Unit, School of Public Health, Imperial College London, London, UK; 4 Munich Cluster for Systems Neurology (SyNergy), Munich, Germany; 5 German Center for Neurodegenerative Diseases (DZNE) Dresden, Dresden, Germany; 6 Center for Regenerative Therapies Dresden (CRTD), Technische Universität Dresden, Dresden, Germany; 7 Sackler School of Medicine, Tel- Aviv University, Ramat Aviv, Israel; 8 Institute of Health and Society, Newcastle University Institute for Ageing, Newcastle University, Newcastle, UK; 9 MRC Biostatistics Unit, Cambridge University, Cambridge, UK; 10 Department of Epidemiology, Erasmus MC University Medical Center, Rotterdam, The Netherlands; 11 Department of Social Medicine, Psychiatry and Neurology, 1st Department of Neurology, Aeginition University Hospital, National and Kapodistrian University of Athens, Athens, Greece; 12 Cognitive Neuroscience Division, Department of Neurology and The Taub Institute for Research on Alzheimer’s Disease and the Aging Brain, Columbia University Medical Center, New York, NY USA; 13 Université Normandie, Inserm, Université de Caen-Normandie, Inserm UMR-S U1237, GIP Cyceron, Caen, France; 14 Institute for Stroke and Dementia Research, University Hospital, LMU Munich, Munich, Germany

**Keywords:** Alzheimer’s disease, Parkinson’s disease, neuroimaging, biomarkers, risk factors, animal models, prevention, epidemiology, cognitive reserve, brain reserve

## Abstract

**Background:**

The concept of reserve was established to account for the observation that a given degree of neurodegenerative pathology may result in varying degrees of symptoms in different individuals. There is a large amount of evidence on epidemiological risk and protective factors for neurodegenerative diseases and dementia, yet the biological mechanisms that underpin the protective effects of certain lifestyle and physiological variables remain poorly understood, limiting the development of more effective preventive and treatment strategies. Additionally, different definitions and concepts of reserve exist, which hampers the coordination of research and comparison of results across studies.

**Discussion:**

This paper represents the consensus of a multidisciplinary group of experts from different areas of research related to reserve, including clinical, epidemiological and basic sciences. The consensus was developed during meetings of the working groups of the first International Conference on Cognitive Reserve in the Dementias (24–25 November 2017, Munich, Germany) and the Alzheimer’s Association Reserve and Resilience Professional Interest Area (25 July 2018, Chicago, USA). The main objective of the present paper is to develop a translational perspective on putative mechanisms underlying reserve against neurodegenerative disease, combining evidence from epidemiological and clinical studies with knowledge from animal and basic research. The potential brain functional and structural basis of reserve in Alzheimer’s disease and other brain disorders are discussed, as well as relevant lifestyle and genetic factors assessed in both humans and animal models.

**Conclusion:**

There is an urgent need to advance our concept of reserve from a hypothetical model to a more concrete approach that can be used to improve the development of effective interventions aimed at preventing dementia. Our group recommends agreement on a common dictionary of terms referring to different aspects of reserve, the improvement of opportunities for data sharing across individual cohorts, harmonising research approaches across laboratories and groups to reduce heterogeneity associated with human data, global coordination of clinical trials to more effectively explore whether reducing epidemiological risk factors leads to a reduced burden of neurodegenerative diseases in the population, and an increase in our understanding of the appropriateness of animal models for reserve research.

## Background

The current paper presents the common consensus of the working groups of the first International Conference on Cognitive Reserve in the Dementias, held on November 24–25, 2017, at the Department of Psychiatry and Psychotherapy of the University Hospital of Ludwig Maximilian University, Munich, Germany, and the Alzheimer’s Association Reserve and Resilience Professional Interest Area, which held its last meeting on July 25, 2018, at the Alzheimer’s Association International Conference in Chicago, IL, USA.

Dementia is becoming more prevalent globally, with the associated burden on societies and healthcare systems constantly increasing [1]. Since the approval of the cholinesterase inhibitors and memantine more than two decades ago [2, 3], further attempts to develop new drugs for dementia have failed. Conversely, research and development efforts in other fields of medicine, such as cancer, have been more successful, primarily due to more advanced approaches that utilise the power of large cohorts to identify new study endpoints and drug candidates [4]. Therefore, a cultural transformation of the dementia research field is urgently required to replicate successes in other disease areas [5].

**Fig. 1:**
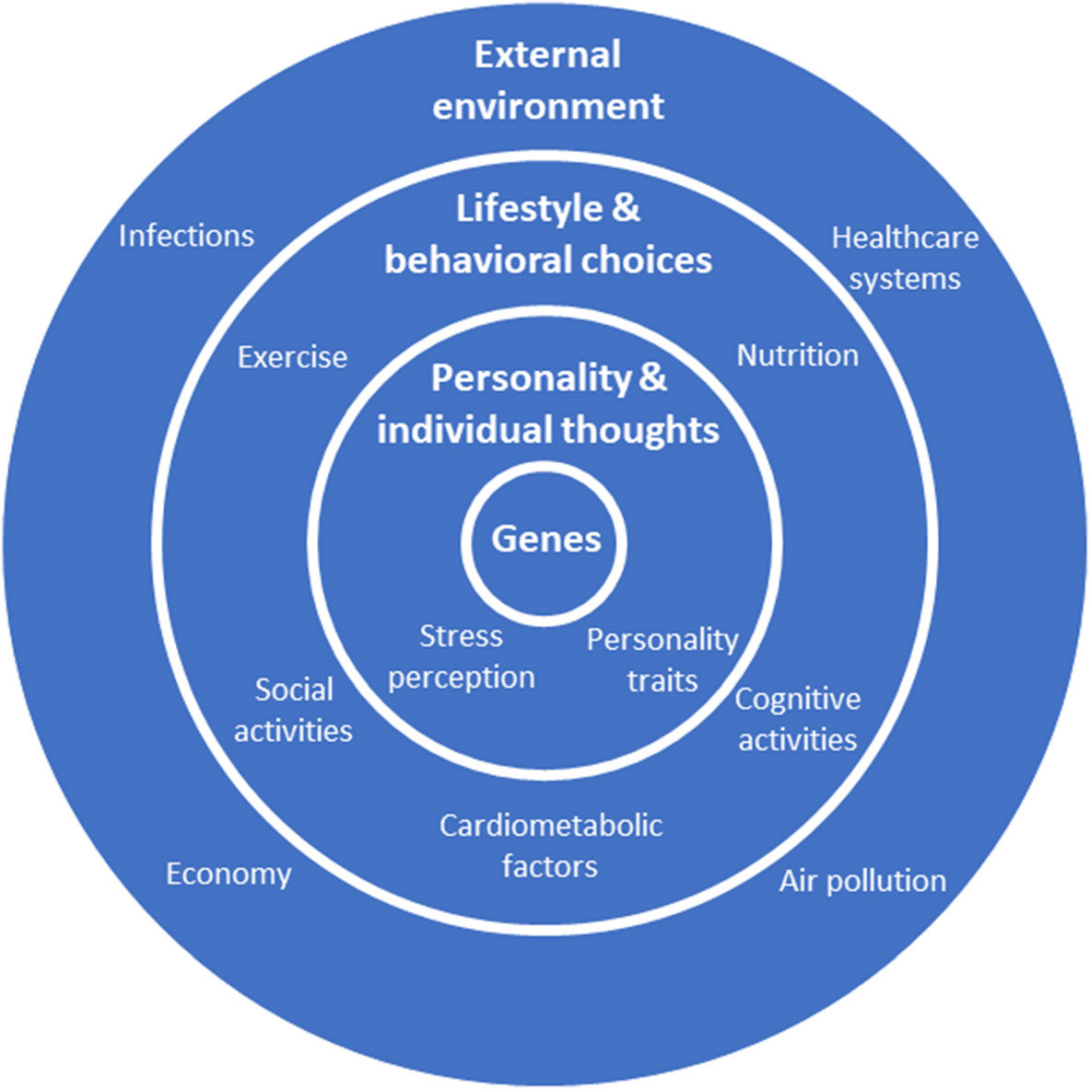
A multicausal model of neurodegenerative dementias (modified from Perneczky [20])

Similar to most other complex diseases, the aetiology of the prevalent neurodegenerative dementias is multifactorial and influenced by a range of diverse parameters such as lifestyle, genetics, an individual’s personality, behavioural decisions and external factors [6]. While genetic susceptibility is largely hereditary and cannot be modified, risk conferred by the environment (including epigenetic mechanisms) can potentially be altered. Indeed, lifestyle changes (e.g. leading to reduced vascular risk) may be an appropriate means to prevent or delay dementia and neurodegenerative changes [7]. Additionally, the role of protective factors is increasingly being recognised, with improved physical and psychological wellbeing through healthier diets and more active lifestyles also likely contributing to dementia prevention. Finally, personality traits (e.g. higher neuroticism) [8] and external factors, such as air pollution and healthcare systems, may also be associated with dementia risk [9] (Fig. 1).

Research on protective factors for different dementias has received growing attention in recent years. A key finding was that higher levels of lifelong experiences, such as cognitive, social and physical activities [10], are associated with a later onset and decreased risk of dementia, which may to some extent explain the reduced age-associated dementia incidence recently reported in some high-income countries [11]. However, the biological mechanisms underlying these protective effects remain largely unknown; improving our mechanistic understanding of these effects is crucial for the development of effective therapies and preventive strategies.

**Fig. 2:**
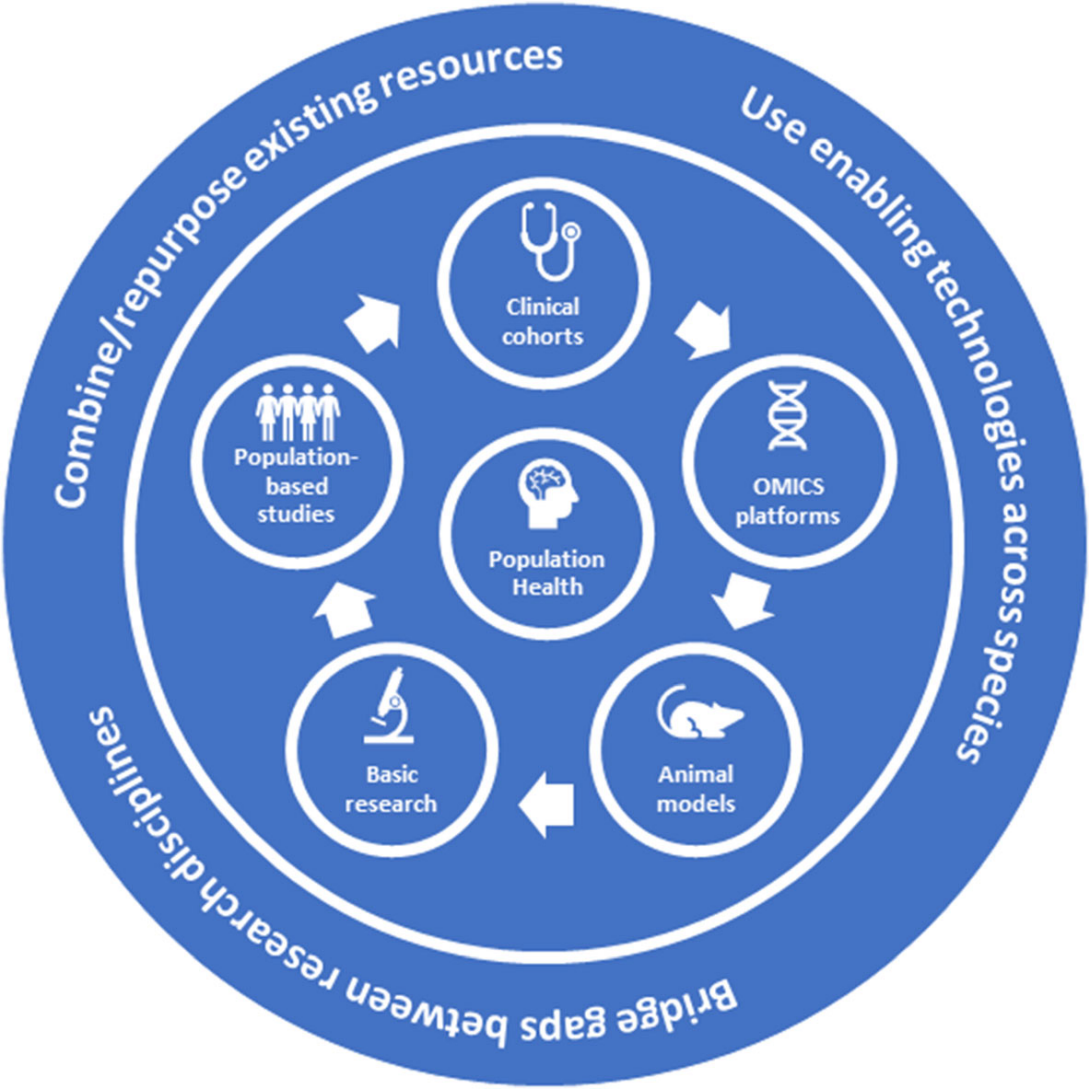
Translational model of reserve against neurodegenerative disease

The main aim of the present paper is to develop a translational perspective on the putative mechanisms underlying reserve against neurodegenerative disease, combining evidence from epidemiological and clinical studies with knowledge from animal and basic science research (Fig. 2). Given the high complexity and multifactorial aetiology associated with neurodegenerative dementias, an approach to study reserve, which integrates the most recent evidence from the relevant disciplines across the traditional boundaries of the different dementia types, seems promising. Herein, we discuss the brain structural and functional underpinnings of reserve as well as relevant genetic factors and lifestyles, both in humans and in animal models. Further, the similarities and differences between different neuropsychiatric disorders such as Alzheimer’s disease (AD), Parkinson’s disease, frontotemporal dementia (FTD), multiple sclerosis (MS) and schizophrenia (SZ) are addressed. Finally, the challenges and opportunities in relation to the design of future observational and interventional studies, with the ultimate aim to strengthen reserve and to improve dementia prevention, are also discussed.

## Conceptual considerations

The concept of reserve was introduced to help explain the disjunction between the degree of observed brain changes or pathology and the clinical manifestation of those brain changes. At this point, three important concepts have emerged, namely brain reserve (BR), brain maintenance (BM) and cognitive reserve (CR) [10, 12, 13]. These concepts were recently defined and discussed in a consensus whitepaper [14].

BR points to the idea that individuals with more neurobiological capital, such as more synapses or neurons, do better in the face of age-related or pathologic brain changes. In the landmark article by Blessed et al. in 1968 [15], the authors noted that six subjects had a dementia score near 0 but a high amyloid-β (Aβ) plaque count. They speculated that “*it would appear that a certain amount of the change estimated by plaque counts may be accommodated within the reserve capacity of the cerebrum without causing manifest intellectual impairment*” [15]. In the context of normal ageing, BR might be considered a brain ‘hardware’ status such as brain volume and white matter integrity. BR has been considered a passive process that simply involves more neurobiological capital. More recently it has been recognised that the brain is much more plastic than we originally imagined. The concept of BM posits that brain integrity does not change in the face of aging and primary pathologies. Many lifestyle factors associated with BR also support BM; it has been suggested that, at any point in time, BR is a function of ongoing BM [12], i.e. BM is defined as a longitudinal concept. Finally, CR focuses on the idea that there are individual differences in adaptability (i.e. flexibility, efficiency, capacity, compensation) of functional brain processes that allow some people to cope better than others with age- and disease-related brain change. Original support for the CR concept came from epidemiologic studies, primarily in AD. There, a reduced risk of incident dementia was noted in individuals with higher educational or occupational attainment [16], premorbid IQ [17] and engagement in leisure activities [18]. The underlying assumption was that all of these groups are comparable in the underlying progression of AD pathology, and that these life experiences influenced individual differences in functional brain processes that, in turn, moderate between pathology and clinical expression. Subsequent studies directly demonstrated that these lifestyle factors moderated between measured AD pathology and cognition [19, 20]. The concept of CR has been successfully applied to normal ageing, as well as to a host of other conditions, including HIV dementia, Parkinson’s disease, MS and head trauma.

It is important for investigators to have consensus regarding the names and meanings of the concepts they use. In this regard, there are ongoing theoretical issues that must be addressed. For example, while BR and CR are useful concepts for research, the conceptual differentiation between brain physiology and its associated cognitive processes is complex. Similarly, there are other concepts used by investigators who explore individual differences that need to be put into context. For example, imaging investigators often explore the phenomenon of compensation, with recent efforts focusing on achieving a consensus on a set of definitions for this concept. While some feel that exploring compensation is one approach to understanding the neural underpinnings of CR, others want to privilege it as a phenomenon in its own right.

It is also useful to remain aware of the context in which these concepts were developed. The concepts of BR and CR were initially put forward in the context of pathologic changes such as AD and were later extended to ‘normal’ age-related changes. In contrast, the concept of BM has primarily been associated with age-related brain changes. Epidemiologic studies of ageing have provided mixed results with regards to the moderating effect of factors such as education on the lifetime rate of cognitive decline and there is evidence that early- versus late-life education may have different effects in relation to reserve [21]. More careful delineation of the brain changes associated with age-related cognitive decline may allow for more direct documentation of the presence of CR, where CR proxies moderate between these brain changes and cognition.

## Epidemiology and genetics

A series of risk and protective factors have been reported to alter the risk for cognitive decline, mild cognitive impairment or dementia, conceivably via reserve-related mechanisms [22–27]. Occupation, education, literacy, and mental, social and physical activities are some of the most commonly described. With few exceptions, a lower incidence of dementia has been reported in subjects with higher education by most epidemiological studies around the world [16, 22, 23, 28–35]. Education is tightly associated with the ability to perform on neuropsychological testing, which is the main instrument used to diagnose dementia; within a reserve framework, it has been hypothesised that education may modify the association between underlying neuropathology and levels of cognitive function [36]. However, individuals with more formal education may simply perform better on certain neuropsychological tests, and this potential circularity must be recognised when using education as a proxy measure for reserve.

Both education and literacy (or IQ) reflect not only genetically determined but also environmental factors [37–39]. However, literacy may be a better marker for educational experiences during the lifetime than formal education for subjects who did not have the chance to receive formal education or to obtain an occupational status corresponding to their abilities [40]. For example, some individuals who were raised during war or are immigrants or minorities may have important intellectually and psychologically demanding roles in their communities, yet this status may not be reflected in years of schooling or occupational attainment. Similarly, in many non-western countries, for instance, in Africa and Asia, the number of years of formal education received is heavily influenced by (parental) income and is therefore a poor marker of the actual reserve accrued through multilinguistic abilities, for example. In addition, because not only years of education but also quality of education and non-formal education during the lifetime may differ, measures of literacy may provide a more meaningful proxy of reserve and should therefore be included in the list of environmental-epidemiological factors affecting risk for dementia. Lower linguistic, cognitive or mental abilities (in some studies estimated quite early in life [41]) have been associated with heavier neurodegenerative pathology burden at autopsy [42], poorer cognitive function in midlife [43], faster rates of cognitive decline [40, 44] and higher dementia rates in late life [45, 46]. It was also suggested that bilingualism is associated with a lower dementia risk and that speaking two or more languages delays the diagnosis of AD by almost 5 years [47, 48]. Bilingual dementia patients were shown to have greater brain atrophy compared to monolinguals, indicating that they had required more severe neurodegeneration to develop symptoms [49]. However, a recent systematic review and meta-analysis suggested that the protective effects of bilingualism were more likely to be found in retrospective compared to prospective studies, indicating that confounding effects of factors such as education may play a role [50].

Neuronal plasticity and development are by no means confined to early life but may be affected in particular by occupational experiences that occupy such a large percentage of our time, energy and effort during adulthood. It has been theorised that reduced everyday experiences and activity patterns may result in disuse and consequent atrophy of cognitive processes and skills (a view captured in the adage ‘use it or lose it’) [51, 52]. In other words, everyday cognitive experience may affect reserve in a manner that is analogous to physical exercise for musculoskeletal and cardiovascular reserve functions. Many studies have reported associations between occupation characteristics and cognitive decline risk [16, 22, 35, 43, 46, 53, 54]. Similarly, population-based research has provided ample support for both intellectual [28, 43, 55–63] and social [28, 53, 64–67] activities in relation to protection from future cognitive decline.

In addition to cognitive activity, there is also evidence for protection by non-cognitive activities. Many studies have reported that physical activities provide protection against future cognitive decline [43, 68–73] and reduced risk of dementia [74–77], including studies showing effects on biomarkers related to physical activity interventions [78, 79]. Decreased risk for cognitive decline has been reported not only for strenuous [70] but also for moderate physical activities [73, 75]. In fact, it has been postulated that motor function has a reserve component as well [80].

Other non-environmental factors potentially affecting reserve may be related to individual genetic or epigenetic characteristics. It should be noted that, although some life experience factors that affect reserve are considered environmental, it is possible that they may reflect some genetic effects; indeed, single nucleotide polymorphisms (SNPs) associated with intelligence [81] and education [82] have been reported. Head size or intracranial volume is another factor related to reserve that is both related to the (perinatal) environment [83–85] and genetic variation [86]. Many of the recent genetic discoveries pertaining to reserve have been achieved by genome-wide association studies (GWAS). An important feature of such GWAS has been that increasing sample sizes have resulted in the discovery of an increasing number of SNPs (e.g. compare [86–89]), thereby further unlocking the genetic underpinnings of reserve. In coming years, the advent of major biobank studies, such as the UK Biobank and German National Cohort, will further boost these numbers.

Besides the many genetic loci discovered for reserve-related phenotypes through GWAS, two important features additionally stand out. First, genetic correlation testing has confirmed that reserve shares a substantial genetic basis with dementia [87, 90, 91]. Second, some genetic variants linked to both reserve, and ultimately dementia, seem to exert their effect already early in life [92], possibly even in utero [87]. Therefore, to develop effective preventive and therapeutic strategies it is pivotal to understand the mechanism from gene through reserve all the way to dementia and to do so across the entire life-span, starting ideally prenatally.

Another relevant point is that most of the epidemiological reserve-related factors are not independent but are rather inter-related. For example, literacy is partially genetically determined but it is also strongly affected by educational experiences, social factors and other environmental factors. For most, education is not strictly environmental since subjects with higher intelligence usually complete more years of schooling [43]. Occupational status is related to education, literacy and socioeconomic factors but also represents a form of non-formal education. Lifestyles and patterns of intellectual, social and physical activities are related to educational and occupational attainment and at the same time represent a life-long type of training. This further emphasises the need for longitudinal life course studies that accurately capture these variables from birth and onward.

Twin studies indicate that many lifestyle attitudes, such as eating patterns [93], smoking [94, 95], sports participation and daily physical activity [96] and even religiosity [97, 98], might be influenced by genetic factors. Therefore, although many of the epidemiological factors affecting reserve are usually examined separately in the scientific literature, they most likely represent convergent or divergent constructs to some degree. Some further factors (i.e. nutrition [99] and others), for which there is strong epidemiological support for associations with risk of cognitive decline and dementia, have not yet been investigated within a reserve-type research framework, e.g. exploring their mediating effects on the association between disease-related brain changes and symptoms due to those changes.

## Reserve in non-Alzheimer’s disease disorders

The question of whether reserve is specific for a given disease or whether it is a phenomenon that can be observed across different disorders is of prime importance both theoretically and in practical terms. Most human data on reserve in relation to cognitive decline comes from epidemiological studies of people with late onset dementia, most of whom have a combination of AD-type pathology and cerebrovascular changes.

In vascular dementia, a population-based study in different cohorts showed that higher education was associated with a risk reduction of dementia due to stroke, indicating that education confers reserve and attenuates the impact of stroke on cognitive function [100]. Bilingualism, another factor associated with higher presumed reserve, was also reported to be associated with better cognitive function after stroke [101]. However, it needs to be cautioned that, in subjects with higher education, a healthier lifestyle is more frequently found, and thus the individual contribution of different reserve proxies is difficult to estimate. In small vessel disease, higher education attenuated the association between white matte damage and cognitive function [102, 103].

There is also a growing body of evidence on the positive effects of protective environmental factors in different non-AD neurodegenerative disorders. FDG-PET studies show that the negative impact of glucose metabolic deficits on cognitive performance is attenuated by years of schooling in AD [104, 105], behavioural variant FTD [106], primary progressive aphasia [107] and dementia with Lewy bodies [108]. The observation that metabolic deficits have a smaller effect on cognitive function in men compared to women (i.e. evidence for a sex-specific component of reserve) has also been reported for different neurodegenerative disorders, including AD [109] and behavioural variant FTD [110]. Taken together, these studies suggest that certain aspects of reserve may be independent from the underlying type of neurodegenerative pathology.

Outside the field of prototypical neurodegenerative disorders, reserve has also been studied in other brain conditions, in particular in MS. Environmental protective factors, including a combination of educational attainment, premorbid IQ and the participation in cognitive leisure activities, were found to have a beneficial role in preserving cognitive function and to moderate the effect of structural brain damage on cognitive performance [111], which is a repeated finding across several studies [112]. Personality traits were also studied in MS in relation to reserve and it was reported that conscientiousness had a synergistic positive effect with childhood enrichment activities on cognitive processing speed [113].

Evidence on the effects of reserve outside of the AD field also exists for SZ. It was shown that higher reserve (estimated by a combination of premorbid IQ, educational-occupational level and leisure activities) was associated with better cognitive (working memory and attention) [114] and functional [115] outcomes after a 2-year follow-up in individuals with a first episode of SZ, controlling for the influence of clinical psychopathology. Environmental protective factors (education-occupation, leisure activities) and premorbid IQ were also related to better neuropsychological and psychosocial function in euthymic patients with bipolar disorder cross-sectionally [116, 117], further underpinning the notion that reserve is not an AD-specific phenomenon.

## Preclinical research and small animal studies

Despite a large and influential literature on the effects of ‘environmental enrichment’ on the brain [118–120], the ideas of BR, CR and BM, which are implicit or even explicit in these experimental studies on mice and rats, have not yet been extensively discussed in basic neurobiological research; interdisciplinary comparative research is essentially absent. The consequence is that the neurobiological foundation of the various types of reserves that have been described often remains vague.

The exact morphological correlates of changes detected in imaging studies are often not known and can only be inferred. For example, determining white matter integrity as a variable in MRI studies [121] does not allow specific conclusions about the microstructure of axons and myelin sheets, including their biochemistry and physiology, which would require microscopy. Conversely, an experimental study on the plasticity of axons will never inform about large-scale patterns of connectivity that MRI is able to assess. Nevertheless, results from basic research on the effects of physical activity or environmental enrichment in animal models are often extrapolated to the human situation and clinical context without considering the limits of the analogy. These findings imply that both lines of research can inform and inspire each other.

A main strength of animal studies in this domain is that the genetic background can be controlled and the environmental stimuli precisely dosed [122], allowing the study of fundamental questions of gene-environment interactions and increasing the likelihood of developing mechanistic theories at the level of genes, signalling molecules, synapses and cells. Such a reductionistic approach is necessary to condense the immense complexity of reserve phenomena with respect to both gaining profound and complete mechanistic insight and developing strategies to improve reserve formation in the medical context [123]. The challenge remains on how to transition from the reduced experimental situation to the full depiction of individual human life. Additionally, the degree of cognitive changes that can be observed in rodents is quite small, further limiting the potential to highlight substantial effects.

Adult hippocampal neurogenesis is a prime example for brain plasticity. Within the mammalian brain, adult neurogenesis in the hippocampus is an exception as other brain regions do not show the lifelong generation of new neurons [124]. The hippocampus as a key structure for memory formation, including autobiographic memory, is often affected early in neurodegeneration and dementia and is one of the best-studied brain regions. New neurons do not contribute to hippocampal function by allowing learning per se but by contributing to the flexible integration of new information into pre-existing contexts and the contextualisation of new information [125, 126]. Importantly, adult hippocampal neurogenesis is regulated by behavioural activity [124], which creates the unique opportunity to study the dynamics and mechanisms of a process of (cellular) brain plasticity from genes and cells to behaviour, including the relevant feedback loops.

The proposed ‘neurogenic reserve’, which describes how an activity-dependent build-up of a potential for neurogenesis maintains lifelong cognitive flexibility and adaptability, does not replace or explain reserve formation and maintenance in other contexts but can still serve as a role model [127]. In contrast, cortical synaptic changes as a substrate of plasticity, and hence reserve, will be much more widespread but also more diffuse, less straightforward to study and even more variable in their contribution to net effects. In the case of adult hippocampal neurogenesis, synaptic plasticity converges on the newly formed cells in a highly defined network situation that provides identifiable and relevant functionality [128].

Controlling both genetic background and the external environment is possible in animal studies and allows addressing the impact of the so-called ‘non-shared environment’, namely the aspect of non-genetic factors that drive brain plasticity according to individual behaviour or exposure; thus, with adult neurogenesis as a primary exemplary readout, ‘enriched environments’ can be developed into an experimental paradigm that captures the biological essence of how an individual’s fate can be shaped. Adult neurogenesis remains an intriguing, albeit particular, example. What is missing are other equally (or more) detailed examples of activity-dependent plasticity and their resulting feedback loops, which would allow the generalisation across brain structures and functional contexts and the development of solid and broad neurobiologically founded reserve concepts.

## Interventional studies

The identification of potentially modifiable risk factors for AD, and dementia in general, has led to an increased interest in testing non-pharmacological interventions based on lifestyle modification with the ultimate aim to strengthen reserve. An inherent conceptual difficulty in such trials targeting reserve is the necessary time lag between the intervention improving reserve and the ultimate effect in reducing the risk of dementia. This explains the inconsistency across trials that use, as the primary endpoint, either cognitive decline, which does not necessarily need to interfere with one’s daily functioning, or dementia diagnosed by a physician according to a standard set of (clinical) criteria. The search for other surrogate phenotypes as the primary outcome, e.g. imaging, to overcome these concerns has not yet yielded the expected results. Still, interventions targeting reserve have not been entirely disappointing.

Non-pharmacological clinical trials emerged in the early 2000s and included cognitive training, physical exercise or nutritional interventions to reduce important risk factors, for example, related to vascular health. A detailed discussion of these interventions is beyond the scope of this paper and available elsewhere [20, 129, 130]. Additionally, the results were mixed [131–133] and trials progressively evolved towards multi-domain interventions targeting several different lifestyle factors simultaneously, in line with the multifactorial causes of AD. Findings from recent prevention trials suggest that older individuals at increased risk for developing dementia may benefit from multi-domain intervention strategies to some extent; however, the effects of such interventions on cognitive and functional outcomes remain to be well established [134, 135] and some studies have not been able to show any benefit of multi-domain interventions [136, 137]. In addition, it is questionable whether the same interventions can be expected to affect different disorders, for example, AD and FTD. Additionally, the existing data do not allow differentiation between the neuroprotective and symptomatic effects of the interventions. Further biologically rooted concepts are therefore needed.

The emotional and affective dimension of ageing has thus far not been directly targeted in clinical trials. Yet, depression is identified as a risk factor for AD [138], stress is associated with brain (especially hippocampal) deterioration [139], and neuroticism and anxiety are associated with an increased cumulative incidence of dementia [140, 141]. Therefore, mental training for stress reduction and emotion and attention regulation could have a beneficial effect on mental health and well-being in ageing populations, and particularly in the reduction of risk or delaying the onset of dementia.

Meditation practice is used to exemplify the potential benefits of an intervention aiming to reduce stress. Studies on this topic are scarce and have limitations [142], but they indicate that meditation tends to have a positive impact on attentional and memory capacities [143], which are particularly relevant in the context of ageing, AD and reserve. Similarly, the effects of meditation on brain structure and function in young adults are particularly marked in frontal and limbic structures, the anterior cingulate cortex and insula [144, 145], all of which are brain regions particularly sensitive to ageing and AD and/or known to be involved in reserve-related mechanisms [146–149].

In ageing, one previous study showed a less marked grey matter volume reduction with age in meditation practitioners compared to controls [150], and a pilot study reported higher brain volume and glucose metabolism in meditators versus controls in areas of the temporo-parietal and prefrontal cortex, insula, and posterior and anterior cingulate [151], highlighting that mediation may offset the impact of age-associated changes on brain function and structure, potentially leading to reduced dementia risk. However, these observations and assumptions will have to be experimentally confirmed in clinical trials before firm conclusions can be drawn.

## Putative functional brain mechanism

Although several protective environmental factors that support reserve have been identified [24], the underlying brain properties are not clear. Many investigators have used functional imaging in order to address this issue. An early paper suggested the study of potential neural implementations of CR, neural reserve and neural compensation [152]. Neural reserve refers to the cognitive networks that are present in young people, and which are influenced by ongoing life exposures. Thus, over time, the efficiency, capacity or flexibility of these network changes, and individual differences in these networks might constitute one implementation of CR. When the brain is challenged by age- or disease-related changes, those with more neural reserve would be able to maintain function more easily. While the concept of neural efficiency was developed in the context of imaging studies, it is closely aligned with the Scaffolding Theory of Ageing and Cognition model, proposed as a concept of cognitive ageing that integrates evidence from structural and functional neuroimaging to explain how the combined effects of adverse and compensatory neural processes produce varying levels of cognitive function [153]. Neural compensation refers to alterations in the way that tasks are brought on by cerebral changes due to ageing or disease that would not be typically seen in a healthy individual. Higher CR could be associated with the ability to recruit a compensatory network, or alternatively, by the lack of the need to recruit this network.

Other conceptual models of compensation as a neural mechanism contributing to reserve have been offered, where any putative compensatory brain mechanism should show a time-dependent quadratic change during disease progression, with an initial increase in brain activity and subsequent decline [154, 155]. These aspects of compensation were mathematically formalised so that the model becomes parameterised and testable [154], and then applied to functional brain changes in Huntington’s disease, an autosomal dominant disease associated with decline in motor and cognitive functions [156, 157]. Those results highlight the power of predictive models to uncover functional brain changes that support reserve.

Most of the studies of the neural implementation of CR have used task-related activation paradigms. Often, they have focused on the relationship of CR proxies to differential efficiency, capacity or flexibility of brain networks during task performance or to compensatory recruitment. Since CR moderates between brain changes and cognitive/clinical status, many studies have incorporated structural measures and assessments of Aβ and/or tau pathology in addition to functional measures. For example, in one study of cognitively normal older adults with negative Aβ scans [158], higher education was related to greater volume and metabolism in the anterior cingulate. Resting functional MRI (fMRI) connectivity analysis showed greater connectivity as a function of education between the anterior cingulate, hippocampus and posterior cingulate, which was in turn associated with better memory and executive function. The authors concluded that reinforcement of the connectivity of the anterior cingulate cortex with distant cortical areas of the frontal, temporal and parietal lobes appears to be an underlying mechanism for education-related reserve in healthy elders.

More recent studies have identified a functional brain substrate that attenuates the association between AD brain pathology and cognitive impairment. A hypothesis-based set of studies focused on the cognitive control network as a putative network supporting reserve. Several fMRI studies showed that a fronto-parietal cognitive control network, in particular a hub in the left frontal cortex (BA44/6 in the Broca area), is related to higher general cognitive performance in young subjects [159, 160]. The flexibly of this control network allows it to adapt its activity to task demand [161]. In a series of resting-state and task-fMRI studies in ageing and different disease stages of AD, it was demonstrated that higher connectivity of that hub in the left frontal cortex (BA44/6) was related to higher reserve [162–165]. In particular, at a higher resting-state left frontal hub connectivity, the association between lower parietal FDG-PET metabolism and episodic memory impairment was ameliorated in sporadic and autosomal-dominant AD [162, 166, 167].

The association between specific cognitive functions/domains, certain brain pathologies and reserve-related functional brain mechanisms also requires testing. Population-based research suggests that CR may help compensate for the effects of pathological changes across individual cognitive functions. In line with these findings, a ‘task-invariant’ CR network, which is activated during several different cognitive tasks, was recently described. A multivariate network which is active during different cognitive tasks, and which also correlates with IQ (a surrogate measure of CR) was derived [168]. The activation of this network also explained some of the performance in fluid reasoning, which was not exclusively related to brain structural characteristics such as cortical thickness. Another future step is to combine different dimensions of putative brain changes, such as regional grey matter volume and fibre tract-based structural connectivity, with functional brain changes to establish a fully integrated model of neural mechanisms underlying reserve.

## Conclusions

The dementia field has undergone a substantial change in recent years. Traditional clinical disease models are being transformed to more biologically oriented classifications [169]. These changes are fuelled by the urgent desire to develop disease-modifying treatments, which require pathophysiological targets to be effective. The combination of national biobank and cohort resources with innovative analytics is a promising approach towards this goal. An important finding of epidemiological research is that dementia and ageing are intimately related processes, both of which lead to the progressive accumulation of organ damage and detrimental biological changes.

There is ample evidence that AD (co-)pathology is the most prevalent pathological change in older individuals with dementia, and there is a credible link between AD-type pathology and cognitive/clinical decline. However, studies also show that this relationship is weaker in the eldest elderly [170]. The assumption of clear-cut dementia subtypes is put into question by biomarker and neuropathological research suggesting that a substantial proportion of clinically ‘pure’ AD cases have mixed pathology at autopsy (e.g. additional cerebrovascular lesions) and that Aβ is commonly found in cognitively normal older adults [171].

Population-based research stresses the importance of environmental and lifestyle-related factors in the complex risk structure of dementia. Lifestyle characteristics appear to be particularly relevant if they are in effect during middle age and many of those factors are associated with vascular health [172]. Reduced vascular burden, better educational systems and other beneficial societal changes during the last 20 to 30 years may underlie the repeatedly reported decreasing age-associated dementia prevalence and incidence in high-income countries [173, 174] versus rapidly increasing dementia numbers in lower-income countries [175]. Vascular disease probably explains a significant part of dementia risk in individuals older than 75 years and pathologies in older people are likely mixed in most instances [176].

The improved knowledge about the epidemiological risk structure of dementia has so far not been successfully translated into effective dementia prevention programmes and there is an ongoing debate about the causality of important risk factors [177, 178]. However, there still is value in identifying which lifestyle and medical factors are potentially modifiable and general practitioners should play a central role in promoting lifestyle changes likely to reduce dementia risk in the population. Despite the implementation of national campaigns, the awareness of links between health-related behaviour and dementia risk is low in the general population [179], and only little is known about the knowledge of primary care staff on risk factors or to what extent such topics are discussed with patients [180]. The overlap with cardiovascular and diabetes risk suggests that dementia should be added to existing chronic disease prevention initiatives already located in primary care.

To implement more effective dementia prevention programmes globally, the fragmented population-based research landscape needs to be more closely aligned, key relationships between societal, psychological and biological risk factors for dementia have to be explored in more detail, and research has to cross the borderlines between the traditional dementia types and aetiologies. A considerable movement across scientific domains and geographical areas to collect longitudinal data and establish comprehensive data repositories for information sharing is currently underway. Projects such as the Health and Retirement Study at the University of Michigan (https://hrs.isr.umich.edu/), which prospectively surveys a representative sample of over 20,000 older Americans, will offer valuable multifaceted information to address key questions about the challenges, but also the opportunities, of ageing. Platforms such as the Gateway to Global Aging (https://g2aging.org/), the Global AD Association Interactive Network (http://www.gaain.org/) and the European Medical Information Framework (http://www.emif.eu/) offer the required infrastructure to pool population surveys and patient-level data to support innovative, large-scale research. These and other similar multinational efforts help to harmonise data across the individual studies and to foster collaboration and data sharing. These efforts will help to address critical questions in relation to clinical, genetic, omics and other data, which are also relevant for research on reserve and which can currently not be answered in any single cohort.

### Recommendations and future perspectives

Based on a thorough qualitative review of important aspects of the reserve literature, our group has agreed on a list of key consensus recommendations that, in our opinion, would help research efforts in this field to provide more informative results with more tangible benefits for the affected or at-risk of disease populations. Firstly, it is crucial that the field reaches consensus on conceptual research models to be used when exploring the different concepts of reserve. For CR one must keep in mind that experiences considered proxies of CR moderate between brain and cognitive changes. For example, in imaging studies designed to explore CR, it is important to keep the level of BR in mind. That is, differential task-related activation occurs in the context of measurable important brain variables, for example, including volume, cortical thickness and white matter tract integrity [181]. CR-related activation patterns should optimally moderate between these brain measures and cognition. Concerted efforts to harmonise research in the reserve field have to be increased, including regular expert meetings (e.g. Alzheimer’s Association Reserve and Resilience Professional Interest Area), consensus conferences (e.g. Conference on Cognitive Reserve in the Dementias) and the publication of evidence-based guidelines [14, 182].

Secondly, along with consensus on concept definitions and research approaches is the need for replication of research findings in ‘test bed’ datasets. For example, if a particular resting fMRI pattern is considered a candidate for a neural representation of CR, it optimally should moderate between brain and cognitive measures. It would be ideal to have standard, shared sets of data that could be used for these types of analyses. Thus, as groups begin to collect extensive longitudinal datasets, establishing opportunities for sharing and for an application of results across datasets will be crucial. The continued improvement of IT platforms, such as the Global Alzheimer’s Association Interactive Network and the European Medical Information Framework, will improve the availability of larger and more diverse sets of data.

Thirdly, advances will be facilitated by collaboration and comparison of results by different laboratories. The substantial heterogeneity of human genetic and environmental factors dictates such collaboration, which can only be achieved after similar methods of data collection and analysis are agreed upon by the research community. Pooling of data into publicly accessible repositories will empower more scientists globally to work on the valuable information sources. Databases such as the International Alzheimer’s and Related Dementias Research Portfolio (https://iadrp.nia.nih.gov/about), which collates and categorises the portfolios of major funding organisations for areas of shared priorities as well as areas of opportunities to inform coordination and collective efforts that seek to advance dementia research, help to coordinate funding strategies and leverage resources in order to maximise the impact on public health and to avoid duplication of effort and reduce inefficiency. However, such efforts will need to be proceeded by the establishment of appropriate ethical, legal and social rules and agreements accepted across regional and cultural boundaries, as advocated by the World Dementia Council (https://worlddementiacouncil.org/our-work/our-work), for instance.

Fourthly, it will also be crucial for studies of the different reserve concepts to move towards longitudinal and, if possible, interventional studies. In these contexts, BM can be directly evaluated and the moderating effects of CR on cognitive or clinical outcomes can be better elucidated. Efforts to translate the knowledge on epidemiological risk and protective factors to clinical research has so far largely been disappointing, and globally coordinated randomised clinical trials are needed to explore if interventions targeting these risk factors can reduce the burden of neurodegenerative diseases in the population [130].

Finally, we need to establish ‘cross talk’ between human and animal studies of these concepts. Experimental neurobiological basic research of the different concepts could improve mechanistic insights. Critical constructs such as maintenance, plasticity and flexibility can be explored at the synaptic, cellular and functional level. This would allow us to approach fundamental questions about the relationship between brain structure and function. In addition, they may supply mechanistic insight into the ideas embodied in the hypotheses of CR, BR and BM. Typically, animal models of AD overexpress human autosomal-dominant genes, which result in overproduction of Aβ. However, these models only capture one, albeit central, aspect of AD pathology and other aspects, such as tau neurofibrils, are not adequately represented [183]. The premature translation of successful animal experiments to human trials has contributed to the high failure rate of AD drug development. Applying knowledge from animal research to human research on reserve carries the same risk of failure. Our understanding of the strengths and weaknesses of each of the different disease models has to improve before we are in a position of successful translational research in the dementia space. The combination of more than one animal model and studies of longer duration to explore relevant disease and reserve mechanisms would help increase the success of translational research. Another challenge of translating findings from animal research to human studies is related to the assessment of cognitive function across the different species. For example, humans are able to categorise and express their memory experience, but it is far more difficult to establish reliable evidence of episodic memories in animals since they are unable to verbally communicate conscious recollection. Equivalent measures of similar cognitive domains in animals and humans are important to improve our understanding of similarities and differences between biological models and human disease. Therefore, developing more appropriate animal cognitive tests is another important prerequisite for successful translational research in the reserve field.

## Abbreviations

AD: Alzheimer’s disease
Aβ: amyloid-β
BM: brain maintenance
BR: brain reserve
CR: cognitive reserve
fMRI: functional magnetic resonance imaging
FTD: frontotemporal dementia
GWAS: genome-wide association studies
MS: multiple sclerosis
SNP: Single nucleotide polymorphism
SZ: schizophrenia
